# Understanding Older Adults’ Intention to Adopt Digital Leisure Services: The Role of Psychosocial Factors and AI-Based Prediction Models

**DOI:** 10.3390/healthcare13070785

**Published:** 2025-04-01

**Authors:** Suyoung Hwang, Hyun Byun, Eun-Surk Yi

**Affiliations:** Department of Exercise Rehabilitation & Welfare, Gachon University, Incheon 21936, Republic of Korea; harriett0059@gmail.com

**Keywords:** older adults, digital leisure, artificial neural networks, digital literacy, technology adoption

## Abstract

**Background/Objective**: As the global aging population grows, digital leisure services have emerged as a potential solution to improve older adults’ social engagement, cognitive stimulation, and overall well-being. However, their adoption remains limited because of digital literacy gaps, psychological barriers, and varying levels of adaptability. This study aims to analyze and predict older adults’ intention to adopt digital leisure services by integrating psychosocial factors, demographic characteristics, and digital adaptability using artificial intelligence (AI)-based predictive models. **Methods:** This study utilized data from the 2022 Urban Policy Indicator Survey conducted in Seoul, South Korea, selecting 2239 individuals aged 50 years and above. A two-step clustering approach was employed: hierarchical clustering estimated the optimal number of clusters, and K-means clustering finalized the segmentation. An artificial neural network (ANN) model was applied to predict the likelihood of digital leisure adoption by incorporating demographic and psychosocial variables. Logistic regression was used for validation, and model performance was assessed through accuracy, precision, recall, and F1-score. **Results:** Four distinct clusters were identified based on digital adaptability and social media engagement. Cluster 3 (highly educated males in their 60s with family support) showed the highest probability (84.35%) of digital leisure adoption despite low social media engagement. Cluster 4 (older women with high social media usage) exhibited lower adaptability to structured digital services. The ANN model achieved an overall classification accuracy of 85.2%, highlighting digital adaptability as a key determinant for adoption. **Conclusions**: These findings underscore the need for targeted policy interventions, including tailored digital education programs, intergenerational digital training, and simplified platform designs to enhance digital accessibility. Future research should further explore psychological factors influencing digital adoption and validate AI-based predictions using real-world behavioral data.

## 1. Introduction

South Korea’s aging population is rapidly increasing, with individuals aged 65 and older projected to surpass 10 million by 2024, accounting for 20% of the nation’s total population [1]. This rapid demographic shift raises significant concerns regarding the quality of life and well-being of older adults. In particular, the COVID-19 pandemic has exacerbated social isolation and loneliness among older individuals, leading to a sharp decline in their physical activity and social engagement, which adversely affects their mental health and overall life satisfaction [2]. As a result, psychological issues such as depression and anxiety have increased, highlighting the urgent need for proactive interventions to support the mental and social well-being of older adults [3].

To address these challenges, senior welfare centers and other institutions have been providing various leisure programs aimed at improving older adults’ physical and psychological well-being while enhancing social interactions [4]. However, as digital technology rapidly evolves and the demand for contactless services grows, leisure services for older adults are also transitioning to digital platforms. This transformation presents both opportunities and challenges, particularly for older adults with limited digital literacy and technological adaptability. Many seniors struggle with digital literacy, face technological accessibility issues, and exhibit psychological resistance toward digital engagement, which hinders their ability to effectively utilize digital leisure services [5,6]. This digital divide exacerbates disparities in access to leisure and health resources, underscoring the need for targeted policy interventions to bridge the gap.

Digital leisure services extend beyond mere entertainment, playing a crucial role in fostering social interaction, cognitive stimulation, and physical health management [7]. Thus, understanding the intentions of older adults to use digital leisure services and identifying the psychosocial factors influencing their adoption are essential for developing effective policies and intervention strategies. The Theory of Planned Behavior (TPB) posits that an individual’s behavioral intentions are shaped by three key factors: attitude, subjective norms, and perceived behavioral control [8]. Similarly, older adults’ adoption of digital leisure services is likely influenced not only by their technological adaptability but also by psychological factors such as motivation, perceived usefulness, and social support from family and community networks [6,9].

Previous research on older adults’ adoption of digital leisure services has predominantly relied on regression and time-series analyses. However, these traditional statistical models often fail to capture the complex and nonlinear nature of consumer behavior. In contrast, artificial neural networks (ANNs) and cluster analysis offer more advanced data-driven methodologies capable of identifying intricate patterns and classifying older adults into distinct segments based on their digital adaptability and behavioral tendencies [10]. This approach enables a more precise understanding of the key barriers to digital leisure service adoption and facilitates the development of targeted interventions tailored to specific user groups.

**Figure 1:**
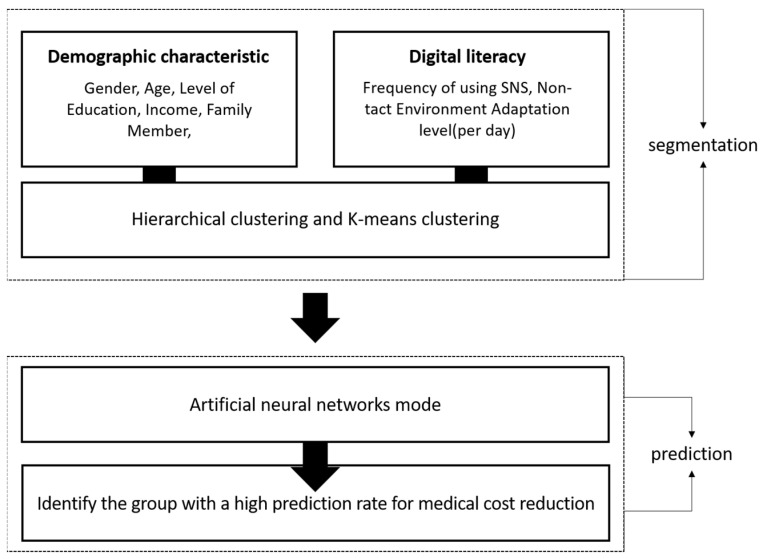
Research design and purpose.

Therefore, this study aims to analyze and predict older adults’ intentions to adopt digital leisure services by integrating psychosocial factors, demographic characteristics, and digital adaptability. Utilizing artificial neural network modeling and cluster analysis, this research employs data from the 2022 Urban Policy Indicator Survey conducted in Seoul. By segmenting the older adult population into meaningful clusters and identifying the key psychosocial determinants influencing their digital service adoption, this study provides strategic insights for policy development. The overall research framework follows a structured approach incorporating hierarchical clustering, K-means clustering, and artificial neural network models to identify groups with a high likelihood of digital leisure adoption (Figure 1). The findings will contribute to enhancing digital accessibility, developing personalized digital literacy education programs, and improving user-friendly digital platforms, ultimately enhancing the quality of life of older adults in the digital age.

## 2. Literature Review

### 2.1. Theoretical Framework: Theory of Planned Behavior (TPB)

The Theory of Planned Behavior (TPB) asserts that an individual’s behavior is primarily determined by their intention, which is shaped by attitudes toward the behavior, subjective norms, and perceived behavioral control [8]. The TPB has been widely applied in studies predicting technology adoption among older adults, demonstrating its effectiveness in explaining behavioral intentions [8,11].

Attitudes toward digital technology play a crucial role in older adults’ willingness to adopt digital leisure services. When older individuals perceive digital services as useful and easy to use, their adoption intentions increase. Conversely, concerns regarding perceived complexity, privacy risks, and lack of relevance often lead to negative attitudes that hinder adoption [12]. Furthermore, subjective norms or the influence of social expectations and family encouragement significantly impact technology adoption, particularly in cultures where intergenerational influence is strong. Older adults may feel more inclined to adopt digital leisure services if their peers or younger family members encourage them to do so [6,9].

Perceived behavioral control also plays a pivotal role in digital adoption, as it reflects an individual’s confidence in their ability to use technology. This factor is particularly relevant for older adults, as digital literacy, prior technological experience, and education level directly influence their self-efficacy in navigating digital environments [13]. Given the importance of these factors, the TPB serves as a comprehensive framework for analyzing the behavioral determinants of digital leisure adoption among older adults.

### 2.2. Older Adults’ Digital Literacy and Cultural Context

Digital literacy, defined as an individual’s ability to access, understand, and effectively utilize digital tools and information, is a significant determinant of older adults’ adoption of digital services. In South Korea, where the digital divide remains a pressing issue, elderly individuals exhibit notably lower levels of digital literacy compared to younger generations. According to the Korea Information Society Development Institute, limited digital proficiency among seniors is a key barrier to their use of digital leisure services. Consequently, targeted digital education programs are essential to bridge this gap and facilitate greater engagement with virtual leisure activities [14,15].

Beyond digital literacy, cultural contexts play a crucial role in shaping technology adoption behaviors. South Korea, as a collectivist society, places a strong emphasis on social ties and familial influence, which significantly affects older adults’ acceptance of new technology. Within this cultural framework, subjective norms and intergenerational support play an instrumental role in mitigating resistance to digital adoption. Older adults who receive encouragement from family members—particularly younger relatives—are more likely to overcome initial reluctance and engage with digital platforms [16].

### 2.3. Psychosocial Factors and Digital Adoption Among Older Adults

Psychosocial factors, including motivation, social support, and perceived behavioral control, are critical in determining older adults’ willingness to engage with digital leisure services. Research has shown that individuals with higher perceived behavioral control—including confidence in using digital tools—are more likely to adopt new technologies. Factors such as prior experience with technology, digital literacy, and educational background all contribute to self-efficacy, influencing adoption behavior [17].

Social support from family, friends, and community networks also plays a key role in technology adoption. Older adults who receive active encouragement, guidance, and digital assistance from their social circles tend to exhibit higher adoption rates. In contrast, those who lack such support may experience greater reluctance and lower digital confidence, reinforcing the digital divide [18]. These findings underscore the necessity of integrating psychosocial and cultural perspectives when analyzing older adults’ digital engagement patterns.

### 2.4. Artificial Intelligence Approaches to Predicting Older Adults’ Digital Adoption

With the advancement of artificial intelligence (AI), predictive modeling has become an increasingly effective tool for analyzing consumer behavior, including digital adoption trends among older adults. Traditional analytical models, such as logistic regression and decision trees, have limitations in capturing the complex, nonlinear interactions between psychological and demographic factors. In contrast, artificial neural networks (ANNs) and clustering techniques offer more sophisticated predictive capabilities [19].

ANN models are particularly effective in analyzing multivariate datasets, as they can identify subtle patterns and interactions that traditional models may overlook. By leveraging deep learning techniques, ANN models can predict digital adoption behaviors with greater accuracy, making them valuable for segmenting older adults based on their likelihood of adopting digital leisure services [20]. Additionally, when combined with cluster analysis, ANN models enable researchers to classify older adults into distinct subgroups, thereby identifying unique behavioral patterns and digital engagement [21].

These AI-based approaches hold significant promise for policy development and service optimization, as they allow researchers and service providers to develop tailored interventions that cater to the specific needs of different user groups. By accurately predicting which older adults are most likely to adopt digital leisure services—and identifying the barriers preventing adoption—policymakers can design more effective digital literacy programs, accessibility solutions, and engagement strategies for older populations.

### 2.5. Summary and Research Gaps

In summary, a comprehensive, interdisciplinary approach is needed to fully understand older adults’ digital leisure adoption behavior. While the TPB provides a strong theoretical foundation for examining psychosocial determinants, and ANN models and clustering techniques offer advanced analytical tools for predictive modeling, existing studies rarely integrate these perspectives. Many previous studies have either focused on socio-psychological factors (e.g., subjective norms, perceived usefulness) or relied solely on traditional statistical methods, overlooking the potential of AI-driven approaches to enhance predictive accuracy.

This study seeks to bridge this gap by integrating psychosocial frameworks with AI-based analytical methodologies, offering a novel approach to understanding and predicting older adults’ digital adoption behaviors among older adults. By combining the TPB, digital literacy assessments, psychosocial factors, and ANN-based predictive modeling, this research aims to develop a more precise and comprehensive framework for facilitating digital leisure adoption among older adults. These insights will provide valuable policy recommendations, helping enhance digital accessibility, literacy programs, and user-centered service design, ultimately improving older adults’ quality of life in an increasingly digital society.

## 3. Materials and Method

### 3.1. Research Design and Participants

This study utilized raw data from the 2022 Urban Policy Indicator Survey conducted in Seoul, South Korea. The survey employed a stratified cluster sampling technique to ensure a representative sample of 5001 citizens aged ≥ 15 years. From this dataset, individuals aged ≥ 15 years were extracted, resulting in a final analytical sample of 2239 participants. This subset was chosen to focus on older adults and pre-seniors, reflecting the study’s emphasis on digital leisure adoption among aging populations. The demographic characteristics of the participants, including gender distribution, age groups, educational attainment, income levels, household size, frequency of social media use, and adaptability to non-contact environments, are summarized in Table 1.

All data collection procedures adhered to ethical research standards and were approved by the Institutional Review Board (IRB) (Approval No. 1044396-202305-HR-084-01).

### 3.2. Measurement of Variables

The dependent variable in this study was the intention to adopt digital leisure services, which was assessed through self-reported survey responses. Participants were asked about their willingness and likelihood of engaging with various digital leisure platforms, including online educational programs, virtual social networking, and digital exercise applications.

The independent variables included demographic characteristics, psychosocial factors, and measures of digital adaptability. Demographic factors included gender, age, educational attainment, income level, and household size. Psychosocial factors were examined through the frequency of social media usage, which was categorized into different levels based on daily usage frequency. Digital adaptability was measured using a Likert scale, where participants rated their level of comfort and confidence in using digital platforms for leisure activities.

Social media usage was categorized based on its frequency, ranging from less than once per day to more than six times per day. Adaptability to virtual environments was assessed using a five-point Likert scale, ranging from “Not adapted at all” to “Very well adapted”, allowing for a more precise measurement of digital competency.

### 3.3. Data Analysis Procedures

#### 3.3.1. Data Preprocessing

The collected data were analyzed using SPSS 23 and IBM Modeler 14.2 software. Cases with missing values, which accounted for less than 3% of the dataset, were handled using listwise deletion, as the missing values were randomly distributed and did not introduce systematic bias. To ensure comparability across variables, Z-score standardization was applied, allowing for accurate clustering and improving model performance.

#### 3.3.2. Clustering Analysis

A two-step clustering approach was employed to segment older adults based on demographic and behavioral characteristics. In the first step, hierarchical clustering was performed to estimate the optimal number of clusters through dendrogram analysis. The results indicate that an appropriate range for clustering would be between four and six clusters. The elbow method and silhouette score analysis were used to determine the most appropriate number of clusters, ultimately identifying a four-cluster solution as the optimal classification.

Following hierarchical clustering, a non-hierarchical K-means clustering method was applied to finalize segmentation. The silhouette score was used to evaluate clustering quality, confirming that the four-cluster solution provided high intra-cluster homogeneity and strong inter-cluster differentiation. This approach ensured that the classification was statistically significant and provided meaningful distinctions between different groups of older adults.

### 3.4. Artificial Neural Network (ANN) Analysis

An artificial neural network model was implemented to predict the likelihood to predict the likelihood of digital leisure service adoption, an artificial neural network (ANN) model was implemented. An ANN was selected due to its superior ability to handle complex, nonlinear relationships compared to traditional statistical models such as logistic regression.

The ANN model architecture was structured to include three hidden layers, which were optimized through systematic testing of different neuron counts, including 1, 2, 3, 4, 8, 16, and 32 neurons per layer. The final model structure used three hidden layer neurons, as this configuration provided the highest classification accuracy while maintaining computational efficiency.

The model was trained using the Quick Propagation algorithm, which enabled rapid convergence during the training process. The sigmoid activation function was applied in the hidden layers, allowing for the effective classification of nonlinear relationships. Meanwhile, a linear activation function was used in the output layer, ensuring precise differentiation between adopters and non-adopters of digital leisure services.

### 3.5. Validation of ANN Model Performance

To ensure the reliability of the ANN model, multiple validation techniques were applied. Logistic regression analysis was conducted as a benchmark comparison, allowing for an examination of the statistical significance of predictor variables and their respective influences on digital adoption behavior.

Several performance metrics were used to assess model accuracy, including precision, recall, F1-score, and confusion matrices. The final ANN model achieved an overall classification accuracy of 85.2%, demonstrating strong predictive power. Precision was recorded at 0.83, recall at 0.79, and the F1-score at 0.81, indicating that the model effectively balanced sensitivity and specificity in predicting digital adoption.

A confusion matrix analysis confirmed that the ANN model had low misclassification rates, further reinforcing its robustness and predictive validity. The inclusion of multiple validation metrics ensured that the model did not overfit and could generalize well to unseen data.

### 3.6. Additional Statistical Analysis

To further investigate the characteristics of each cluster, additional statistical analyses were conducted. Cross-tabulation analysis was performed to examine the relationships between demographic variables and cluster membership, identifying notable differences in age, gender, and educational levels among the groups.

One-way ANOVA was employed to compare the mean differences in digital adaptability and social media usage across the four clusters. The results reveal statistically significant variations, supporting the hypothesis that different demographic segments exhibit unique digital adoption behaviors.

To verify the robustness of the cluster segmentation, Scheffé’s post-hoc test was applied. This test confirmed that all clusters were statistically distinct from one another, reinforcing the validity and reliability of the clustering methodology.

### 3.7. Summary of Methodological Approach

The combination of clustering, ANN-based predictive modeling, and validation through traditional statistical methods enabled a comprehensive and data-driven analysis of older adults’ engagement with digital leisure services. The integration of psychosocial and demographic factors with advanced machine learning techniques allowed for precise behavioral prediction, offering valuable insights for policymakers and service providers.

By applying artificial intelligence-driven segmentation, this study contributes to the development of targeted digital interventions aimed at improving digital accessibility and usability for aging populations. The methodological framework employed in this study provides a robust foundation for future research, particularly in the field of digital inclusion and technology adoption among older adults.

## 4. Results

### 4.1. Cluster Analysis

A two-step clustering approach was employed to identify distinct user groups based on demographic characteristics and psychosocial factors. The hierarchical clustering method was first applied to estimate the optimal number of clusters, which was determined to be between four and six through dendrogram analysis and the elbow method. Further confirmation was obtained through silhouette score analysis, ensuring a high degree of intra-cluster similarity and inter-cluster distinction. Subsequently, K-means clustering was applied to finalize the segmentation and ensure meaningful classification of the participants.

The final clustering solution identified four distinct clusters, each with significant demographic and psychosocial differences, particularly in age, gender, education level, income, and household size (*p* < 0.001). These clusters captured variations in digital adaptability, social media engagement, and family structure, providing a more nuanced understanding of the factors influencing older adults’ digital leisure adoption behaviors. A detailed summary of the cluster characteristics is provided in Table 2. 

### 4.2. Artificial Neural Network Model

**Figure 2:**
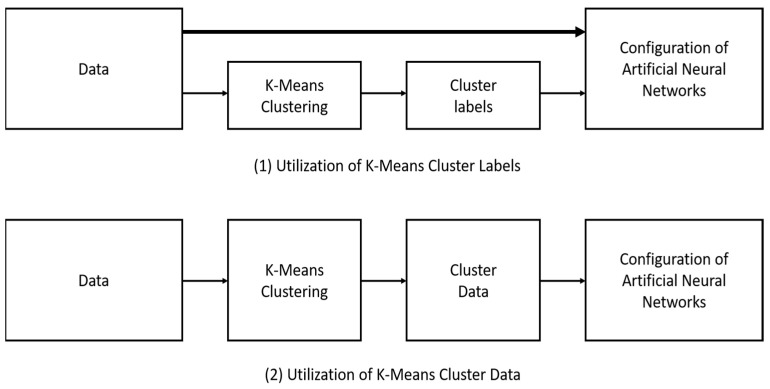
Application of K-means in artificial neural networks.

An artificial neural network (ANN) model was applied to predict the intention to adopt digital leisure services within the identified clusters. The ANN model demonstrated high predictive accuracy, particularly for Cluster 3, which was labeled as ‘Highly Educated 60s Male Group with Family Cohabitation, Low Social Media Involvement, and High Adaptability to Digital Environments’ (Figure 2). This group exhibited the highest classification accuracy (88.8%), suggesting a high likelihood of adopting digital leisure services.

The classification accuracies of other clusters varied. Cluster 1, which comprised ‘Highly Educated, High-Income Men Active in Social Media and Adapted to Virtual Environments’, achieved an accuracy of 83.1%. Cluster 2, characterized as a ‘Low-Educated 60s Male Group with Spousal Cohabitation and Low Social Media Involvement’, recorded an accuracy of 81.1%. In contrast, Cluster 4, consisting of a ‘High-School Educated Female Group with Low Social Media Engagement’, had the lowest classification accuracy at 65.3%.

To further validate the ANN model, classification metrics such as precision, recall, and F1-score were calculated for each cluster. The detailed classification metrics are presented in Table 3, confirming that Cluster 3 exhibited the best overall performance in predicting the likelihood of adopting digital services. These results underscore the importance of educational attainment, family structure, and digital adaptability in influencing older adults’ engagement in digital leisure services.

### 4.3. Cluster Characteristics

Among the identified clusters, Cluster 3 demonstrated the highest likelihood of adopting digital leisure services. This group was primarily composed of educated males in their 60s, living with family members. As detailed in Table 4, 68% of Cluster 3 participants were male, 77.3% were aged between 60 and 69, and 52.9% had a college education. This group also had a high proportion of respondents (61.5%) living in households with more than four members (Table 4).

Despite having low social media engagement (54.3% used social media less than once per day), Cluster 3 members displayed strong adaptability to digital environments, with 46% reporting moderate to high adaptability. These findings suggest that while frequent social media usage was lower, factors such as family support and higher technological adaptability played a crucial role in digital leisure service adoption.

Cluster 1 was identified as a ‘Highly Educated, High-Income Male Group in their 50s, Active in Social Media, and Adapted to Virtual Environment’. In this cluster, 68.8% had a college degree, 46.2% earned over KRW five million per month, and 71.2% reported more than six social media interactions per day. The high level of digital engagement and economic stability in this group facilitated a strong inclination toward digital leisure adoption.

Cluster 2, described as a ‘Low-Educated 60s Male Group with Spousal Cohabitation and Low Social Media Involvement’, exhibited lower educational attainment, with 71.4% having less than a high school education. The participants in this cluster were more likely to be married but displayed limited engagement with digital and social media platforms.

Cluster 4 consisted of ‘High School-Educated 60+ Female Group with High Social Media Usage and Moderate Adaptability’. This cluster had a balanced gender distribution (50.4% female), significant social media involvement (57.1% used social media more than six times per day), and moderate digital adaptability. The contrast between frequent social media engagement and lower adaptability to broader digital environments suggests a narrow scope of digital interactions within this group.

The diverse characteristics of these clusters highlight the need for tailored digital intervention strategies, ensuring that digital leisure services are designed to accommodate the specific needs and digital behaviors of different subgroups of older adults (Table 5).

### 4.4. Validation and Statistical Significance

Additional statistical analyses were conducted to confirm the robustness of the clustering results and predictive models. Logistic regression analysis revealed significant relationships between demographic and psychosocial factors and digital leisure service adoption intentions.

To further verify cluster distinctiveness, cross-tabulation tests and one-way ANOVA were applied, which demonstrated statistically significant differences across key demographic and behavioral variables (*p* < 0.001). These findings indicate that each cluster represents a unique subgroup with distinct characteristics that influence digital adoption behavior.

A post-hoc analysis using Scheffé’s method further validated the statistical significance of the differences between clusters. The test confirmed that each cluster was meaningfully distinct, reinforcing the validity and reliability of the segmentation approach.

Overall, the combination of ANN modeling and cluster analysis provided a comprehensive and detailed understanding of the factors influencing older adults’ digital leisure adoption. These findings offer valuable insights for policymakers and digital service providers, enabling the development of targeted interventions that cater to diverse user needs and enhance digital inclusion among older populations.

## 5. Discussion

This study employed artificial neural network (ANN) models and cluster analysis to examine and predict the intention of older adults to adopt digital leisure services based on their adaptability to virtual environments. These findings provide insights into how demographic characteristics, digital literacy, and social behaviors influence digital service adoption among older adults, particularly in the context of an aging population.

### 5.1. Interpretation of the Findings

The demographic composition of this study aligns with broader trends in South Korea’s aging society. According to national statistics, the average life expectancy in South Korea is 79 years, with women (82 years) outliving men (76 years). This gender disparity explains the higher proportion of female participants in the study. Additionally, the current elderly population consists of individuals who received their education in the 1960s, a period when high school completion was not mandatory. The rapid economic growth during that era also influenced educational attainment levels, resulting in a lower proportion of highly educated individuals among today’s elderly population. Furthermore, family planning policies and increased access to birth control in the 1960s and early 1990s contributed to a reduction in average household size, which has implications for household support structures among older adults today.

### 5.2. The Role of Psychosocial Factors in Digital Engagement

Psychosocial factors play a significant role in digital engagement among older adults. Previous research has shown that older adults who perceive themselves as younger than their actual age are more likely to remain active in social and economic life, including leisure activities [22]. However, prior research has not adequately distinguished between face-to-face and virtual leisure participation. This study addresses this gap by specifically examining the factors that contribute to digital leisure adoption, particularly in a virtual context.

Through hierarchical and K-means clustering, four distinct clusters were identified:

Cluster 1: Highly educated, high-income men in their 50s, familiar with virtual environments and social media.
Cluster 2: Low-educated married men in their 60s with low social media engagement.
Cluster 3: Educated men in their 60s with high virtual adaptability, low social media engagement, and large household sizes.
Cluster 4: Educated women in their 60s actively engage in social media.

Among these groups, Cluster 3, comprising men in their 60s with relatively high education levels and family cohabitation, demonstrated the highest probability (84.35%) of adopting digital leisure services despite lower engagement with social media. These findings suggest that adaptability to virtual environments, rather than social media usage frequency, is a stronger predictor of digital leisure service adoption.

The characteristics of Cluster 3 further reinforce the role of household composition in digital engagement. A significant proportion (61.5%) of individuals in this group lived in households with four or more members. This supports previous findings suggesting that larger family sizes contribute to improved digital skills and higher adoption of digital technologies among older adults [23]. Social and family influences likely assist older individuals in navigating digital platforms. Additionally, the high adaptability to virtual environments across all clusters suggests that older adults are willing to engage with digital leisure services when provided with the necessary tools and support.

### 5.3. The Applicability and Limitations of ANN Models

Although the ANN model demonstrated high classification accuracy, its real-world applicability requires further validation. The predictive power of ANN should be interpreted cautiously, as the study does not incorporate real-world adoption rates of digital leisure services among older adults. Future research should validate these predictions by examining actual behavioral data over time to determine whether individuals classified as high adopters truly engage with digital leisure services. This validation process would strengthen the model’s generalizability and address concerns about the potential overestimation of digital adaptability among specific clusters.

Beyond technical accessibility, psychological barriers such as motivation, perceived usefulness, and self-efficacy also influence digital adoption among older adults. Even when digital access is provided, individuals with low confidence in digital interactions may still hesitate to engage with virtual services. Prior research indicates that older adults with lower perceived usefulness of digital platforms are significantly less likely to adopt digital services, even when accessibility is not an issue. Future studies should explore interventions that improve not only digital skills but also psychological readiness for virtual engagement.

### 5.4. Gender Disparities and Sociocultural Influences

Sociocultural factors may also influence gender disparities in digital adoption. In traditional patriarchal societies, older men may have greater decision-making power over household technology investments, whereas women—particularly those in caregiving roles—may face time constraints that limit their engagement with digital leisure services.

Furthermore, the digital participation rate among older women remains lower than that of older men despite their high engagement with social media (as seen in Cluster 4). This suggests that while older women frequently use digital platforms for social networking, they may lack confidence in adopting broader digital leisure services such as virtual education or exercise programs.

In addition, decision-making regarding technology adoption in male-headed households may contribute to a gender gap in digital participation. As older men are more likely to be household heads, they may have greater control over technology use within the family, further limiting older women’s independent adoption of digital leisure services. This highlights the need for targeted interventions that empower older women by enhancing their digital self-efficacy through structured digital education programs and community-based digital training initiatives.

Developing gender-sensitive digital literacy programs can help bridge this gap by addressing specific barriers that older women face, such as lower confidence in technology use, limited exposure to advanced digital tools, and sociocultural restrictions on independent decision-making. These programs should incorporate interactive training models, mentorship programs, and intergenerational support systems to encourage women’s active engagement in digital leisure services. Additionally, community-based digital literacy initiatives and government-sponsored technology access programs should be explored as potential solutions for reducing digital disparities among older women, particularly those with lower education levels or limited technological exposure.

### 5.5. Policy and Practical Implications

Given these insights, it is essential to focus on enhancing digital accessibility and usability rather than solely increasing social media engagement. Policy and program development should prioritize digital education initiatives that address usability challenges for older adults. The implementation of tailored digital training programs, simplified user interfaces, and intuitive platform designs can facilitate adoption. Moreover, virtual leisure service providers should consider user-centered designs that cater to the cognitive and physical capabilities of older adults.

These findings further suggest that policy strategies for promoting digital leisure engagement should be customized according to different user segments. For instance, while Cluster 1 (high-income, highly educated men in their 50s) may benefit from advanced digital literacy programs, Cluster 2 (low-educated, married men in their 60s) may require more fundamental digital skill training. Similarly, Cluster 4 (older women with high social media engagement) may benefit from programs that transition their existing social media use to productive digital leisure activities.

### 5.6. Future Research Directions

This study highlights the importance of considering digital accessibility and virtual environment adaptability when designing interventions to enhance leisure participation among older adults. Future research should explore additional factors, such as motivation, perceived usefulness, and psychological barriers to digital adoption. Longitudinal studies could provide deeper insights into how digital leisure behavior evolves over time. By implementing targeted strategies and improving digital inclusion efforts, policymakers and service providers can foster greater engagement in digital leisure services, ultimately enhancing the quality of life for older adults.

## 6. Conclusions

This study examined the intention of older adults to adopt digital leisure services by employing artificial neural networks (ANNs) and cluster analysis to assess the influence of demographic characteristics, psychosocial factors, and digital adaptability. The findings highlight the importance of virtual environment adaptability as a key determinant in digital leisure service adoption rather than social media usage frequency alone.

One of the major contributions of this study is the identification of distinct user segments through cluster analysis, providing valuable insights for policy and program development. The results suggest that policy strategies for promoting digital leisure engagement should be customized according to different demographic and behavioral clusters. Given the diverse characteristics of each cluster, a one-size-fits-all approach to digital inclusion may be inadequate. Instead, policy interventions must be tailored to the specific needs and challenges of each user segment to maximize digital engagement among older adults.

Cluster 1, composed of highly educated, high-income men in their 50s, already demonstrates strong digital literacy and frequent engagement with social media. This group may benefit from advanced digital literacy programs that focus on emerging digital trends such as AI-powered platforms, cybersecurity awareness, and blockchain-based leisure services. Collaboration with technology companies and academic institutions could facilitate the development of specialized digital workshops tailored to their interests and technological proficiency. Given their familiarity with digital environments, policymakers should focus on promoting lifelong digital learning programs to keep them engaged with evolving technologies.

Cluster 2, consisting of low-educated, married men in their 60s, shows limited digital skills and low exposure to online platforms. Addressing their digital inclusion requires fundamental digital skill training programs that provide hands-on experience with essential digital tools. Establishing hybrid training programs that combine in-person workshops with gradual online engagement may be an effective strategy for transforming this group into digital leisure activities. Public libraries, community centers, and local government initiatives should play a key role in delivering basic digital literacy courses that cover essential functions such as navigating digital platforms, accessing government services online, and engaging with digital leisure activities.

Cluster 3, which comprises highly educated men in their 60s living with family members, exhibits high adaptability to virtual environments despite low engagement with social media. The strong household support in this group indicates that family members can act as facilitators in digital adoption. Policies that encourage intergenerational digital education, such as family-based digital training programs, could enhance digital engagement within this cluster. Developing incentive-based programs where younger family members assist older adults in adopting digital technologies may further strengthen digital participation. Additionally, government-sponsored tax incentives for households that engage in intergenerational digital education programs could serve as an effective intervention strategy.

Cluster 4, consisting of educated older women with high social media engagement, presents a different set of digital adoption characteristics. While Cluster 4 members actively use social media, their participation in structured digital leisure activities remains limited. To bridge this gap, policy interventions should focus on expanding their engagement by integrating social media with structured learning and health programs.

Leveraging familiar platforms such as Facebook and YouTube to provide online educational courses, virtual fitness programs, and hobby-based online communities could offer a more meaningful digital leisure experience. Additionally, transitioning their existing social media use toward structured digital engagement through tailored educational and wellness programs (e.g., on Facebook or KakaoTalk) may be an effective strategy.

Local governments and private sector partnerships can support this transition by developing online communities tailored to older women’s interests, such as virtual book clubs, fitness programs, or specialized social networking platforms for seniors. Encouraging participation through incentive-based programs and community-driven initiatives can further enhance digital inclusion for this group.

Beyond demographic and technological factors, psychological and social barriers also play a crucial role in determining whether older adults adopt digital leisure services. According to the Theory of Planned Behavior (TPB), perceived usefulness and motivation significantly influence behavioral intention. Older adults who perceive digital leisure services as beneficial and relevant to their daily lives are more likely to engage with them. In contrast, those who view these services as complex or unnecessary may refrain from adoption. Moreover, digital self-efficacy—or confidence in using digital platforms—strongly influences digital engagement. Even if access to digital services is provided, older adults with low confidence in navigating these platforms may be hesitant to participate. This underscores the necessity of addressing not only digital accessibility but also psychological readiness through structured interventions. Digital training programs should not only teach technical skills but also focus on enhancing digital confidence, ensuring that older adults feel comfortable and capable in virtual environments.

The findings of this study further emphasize the need to improve digital accessibility and usability, rather than merely increasing social media engagement. User-friendly digital interfaces should be prioritized to accommodate the physical and cognitive limitations associated with ageing. Digital service providers should incorporate senior-friendly UX standards, such as voice-activated navigation, larger fonts, and simplified interface designs, to enhance usability for older adults. Collaboration with technology developers and policymakers is essential to ensure that digital leisure services are designed with inclusivity in mind.

Additionally, gender-sensitive interventions should be considered, as older men and women experience different barriers to digital adoption due to sociocultural expectations and caregiving responsibilities. In male-headed households, decision-making regarding technology adoption may be influenced by traditional gender roles, potentially limiting older women’s independent engagement with digital services. This highlights the importance of targeted interventions that empower older women by enhancing their digital self-efficacy through structured digital education programs and community-based digital training initiatives.

While this study provides meaningful insights, further research is needed to explore additional psychological factors, such as motivation, perceived usefulness, and self-efficacy, which may influence digital adoption among older adults. Longitudinal studies could offer deeper insights into how digital engagement evolves over time and identify patterns of sustained digital leisure participation. Additionally, examining community-based digital literacy initiatives could help determine the effectiveness of local interventions in bridging the digital divide for older populations.

By implementing targeted strategies and enhancing digital inclusion efforts, policymakers, technology developers, and service providers can foster greater engagement in digital leisure services. In turn, this can contribute to improving the overall quality of life of older adults in an increasingly digital society.

## Figures and Tables

**Table 1:** Demographic characteristics.

Variable	Category	N	%
Gender	Male	1048	46.8
	Female	1191	53.2
Age	50s	869	38.8
	Over 60	1370	61.2
Education Level	Lower than middle school	277	12.4
	Lower than high school	1336	59.7
	Lower than bachelor’s degree	626	27.8
Monthly Income	Less than KRW 500,000	4	0.2
	KRW 500,000 ~ 2 million	170	11.9
	KRW 2 ~ 3.5 million	646	45.1
	KRW 3.5 ~ 5 million	390	27.2
	More than KRW 5 million	222	15.5
Household Size	1	250	11.2
	2	1030	46
	3	498	22.2
	More than 4	461	20.6
Social Media Usage Frequency (per day)	Under 1	523	23.4
	2~3	325	14.5
	4~5	279	12.5
	More than 6	404	18
Adaptability to Digital Environment (per day)	Not adapted at all	33	1.5
	Not very adapted	237	10.6
	Average	910	40.6
	Somewhat well-adapted	832	37.2
	Very well-adapted	227	10.1

**Table 2:** Results of clustering.

Category	Final Cluster				MSE	MAE	F	*p*
	Cluster 1	Cluster 2	Cluster 3	Cluster 4				
Gender	78.4% Male	61.5% Male	68.0% Male	49.6% Male	0.222	1045	14.819	<0.001
Age Distribution	75.5% in 50s	39.1% in 60s	77.3% in 60s	50.8% in 60s	0.197	1045	95.173	<0.001
Education Level	68.8% Bachelor’s or Higher	71.4% High School or Below	52.9% Bachelor’s or Below	69.3% High School or Below	0.269	1045	66.537	<0.001
Income Level	46.2% earning over KRW 5 million	24.6% earning KRW KRW 3.5 million to 5 million	40.6% earning KRW KRW 2 million to 3.5 million	65.1% earning KRW KRW 2 million to 3.5 million	0.516	1045	181.204	<0.001
Household Size	38.9% with 3 members	73.5% with 2 members	61.5% with ≥4 members	60.5% with 2 members	0.392	1045	422.851	<0.001
Social Media Usage Frequency	71.2% using >6 times/day	64% using ≤1 time/day	54.3% using ≤1 time/day	57.1% using >6 times/day	0.278	1045	1486.009	<0.001
Adaptability to Digital Environment	47.1% Somewhat Well-Adapted	51.1% Average Adaptation	46% Somewhat Well-Adapted	45% Average Adaptation	0.637	1045	24.384	<0.001
Number of Cluster Case	208	325	278	238	df = 1045			

**Table 3:** Analysis of predictive probability (classification accuracy) for leisure service use by cluster using an artificial neural network model.

Category		Cluster 1	Cluster 2	Cluster 3	Cluster 4
The number of hidden layer neurons was set to 3 after review.		Leisure service usage	Leisure service usage	Leisure service usage	Leisure service usage
Training	Low probability	0%	0%	0%	0%
	High probability	100%	100%	100%	100%
	All	73.8%	78.7%	79.9%	73.1%
Validation	Low probability	0%	0%	0%	0%
	High probability	100.0%	100%	100%	100
	All	83.1%	81.1%	88.8%	65.3%
Precision		0.81	0.78	0.85	0.71
Recall (Sensitivity)		0.79	0.76	0.87	0.68
F-1 Score		0.80	0.77	0.86	0.69
Confusion Matrix (TP/FP/FN/TN)		162/36/18/284	189/42/21/248	220/28/12/240	140/60/40/200
Average Classification Accuracy		78.45	79.9	84.35	69.2%

**Table 4:** Results of cross-analysis based on demographic characteristics by cluster.

Category		Cluster 1	Cluster 2	Cluster 3	Cluster 4	*x* ^2^	*** *p* < 0.001
Gender	Male	163 (78.4%)	200 (61.5%)	89 (68%)	118 (49.6%)	42.807	0.001
	Female	45 (21.6%)	125 (38.5%)	18 (32%)	120 (50.4%)		
Age	50s	157 (75.5%)	86 (26.5%)	63 (22.7%)	87 (36.6%)	225.106	0.001
	Over 60	51 (24.5%)	239 (73.5%)	215 (77.3%)	151 (63.4%)		
Education Level	Less than middle school	1 (0.5%)	18 (5.5%)	5 (1.8%)	23 (9.7%)	180.233	0.001
	Less than high school	64 (30.8%)	232 (71.4%)	75 (45.3%)	165 (69.3%)		
	Less than bachelor’s degree	143 (68.8%)	75 (23.1%)	147 (52.9%)	50 (21%)		
Income Level	Less than KRW 500,000	0	3 (0.9%)	0	1 (0.4%)	393.654	0.001
	KRW 500,000–2 million	0	44 (13.5%)	8 (2.9%)	50 (21%)		
	KRW 2 million–3.5 million	20 (9.6%)	182 (56%)	113 (40.6%)	155 (65.1%)		
	3.5 million–5 million	92 (44.2%)	80 (24.6%)	105 (37.8%)	31 (13%)		
	More than KRW 5 million	96 (46.2%)	16 (4.9%)	52 (18.7%)	1 (0.4%)		
Household Size	1	3 (1.4%)	39 (12%)	0	39 (16.4%)	632.159	0.001
	2	46 (22.1%)	239 (73.5%)	2 (0.7%)	144 (60.5%)		
	3	81 (38.9%)	47 (14.5%)	105 (37.8%)	45 (18.9%)		
	more than 4	7 (3.4%)	0	171 (61.5%)	10 (4.2%)		
Social Media Usage Frequency	Less than 1	0	208 (64%)	151 (54.3%)	0	997.057	0.001
	2~3	0	117 (36%)	104 (37.4%)	0		
	4~5	60 (28.8%)	0	23 (8.3%)	102 (42.9%)		
	More than 6	148 (71.2%)	0	0	136 (57.1%)		
Adaptability to Digital Environment	Not adapted at all	0	8 (2.5%)	0	3 (1.3%)	82.110	0.001
	Not very adapted	7 (3.4%)	43 (13.2%)	11 (4%)	21 (8.8%)		
	Average	76 (36.5%)	166 (51.1%)	97 (34.9%)	107 (45%)		
	Somewhat well-adapted	98 (47.1%)	84 (25.8%)	128 (46%)	90 (37.8%)		
	Very well-adapted	27 (13%)	24 (7.4%)	42 (15.1%)	17 (7.1%)		

***: *p* < 0.001.

**Table 5:** Comparison and summary of characteristics by cluster.

	Cluster 1	Cluster 2	Cluster 3	Cluster 4
Cluster Description	High-income, educated men in their 50s, familiar with social media, and non-contact environments	Low-educated, married men in their 60s, low engagement with social media	Educated, family-living men in their 60s, low engagement with social media, and good adaptation to non-contact environment	High school-educated, married women in their 60s high engagement with social media
Gender	78.4% male	61.5% male	60.9% male	50.4% female
Age	75.5% in their 50s	39.1% in their 60s	77.3% in their 60s	50.8% in their 60s
Education Level	68.8% college graduates	71.4% high school or below	52.9% college graduates or below	69.3% high school or below
Income Level	46.2% earning over KRW 5 million	24.6% earning KRW 3.5 million to 5million	40.6% earning KRW 2 million to 3.5 million	65.1% earning KRW 2 million to 3.5 million
Household Size	38.9% with 3 members	73.5% with 2 members	61.5% with 4 or more members	60.5% with 2 members
Social Media Usage Frequency	71.2%using social media more than 6 times	64% using social media once or less	54.3% using once or less	57.1% using social media more than 6 times
Adaptability to Digital Environment	47.1% somewhat well-adapted	51.1% average adaptation	46% somewhat well-adapted	45% average adaptation

## Data Availability

The data presented in this study are available upon reasonable request from the corresponding author. The dataset originates from the 2022 Urban Policy Indicator Survey conducted by the Seoul Metropolitan Government under the authority of the Korean government. Access to the data is subject to the Republic of Korea’s data-sharing policies and may require prior approval for secondary analysis.
